# Mechanisms of Mind-Body Interaction and Optimal Performance

**DOI:** 10.3389/fpsyg.2017.00647

**Published:** 2017-05-09

**Authors:** Yi-Yuan Tang, Brian Bruya

**Affiliations:** ^1^Department of Psychological Sciences, Texas Tech UniversityLubbock, TX, USA; ^2^History and Philosophy Department, Eastern Michigan UniversityYpsilanti, MI, USA

**Keywords:** mind-body interaction, optimal performance, mindfulness meditation, integrative body–mind training (IBMT), anterior cingulate cortex, striatum, central nervous system (CNS), autonomic nervous system (ANS)

Studies have long indicated that effort increases focus on the attentional target and increases distraction inhibition and this type of cognitive control enhances performance (Kahneman, 1973). However, more evidence also shows that a reduction in effortful control can also improve performance, such as in creativity, implicit learning and sensorimotor skills, consistent with the multi-action plan (MAP) model in sport performance (Beilock et al., 2002; Bortoli et al., 2012; Ding et al., 2014a; Stillman et al., 2014; Bertollo et al., 2015, 2016; Amer et al., 2016). We use one form of mindfulness meditation - integrative body-mind training (IBMT) in our series of randomized studies. IBMT emphasizes no effort or less effort to control mind and opening awareness to internal and external stimuli with an attitude of acceptance and equanimity. Our results show that as few as 5 sessions of IBMT (20–30 min per session) can improve attention, positive emotion and diverse cognitive performance including creativity, working memory, conflict resolution and learning (Tang et al., 2007, 2014, 2015; Posner et al., 2010; Ding et al., 2014a,b; Fan et al., 2014, 2015; Tang, 2017). This raises the possibility that less effortful attention or effortless attention can contribute to performance in activities involving creativity, sensorimotor skills or implicit learning.

Based on recent findings, we propose a framework for a relationship among attention, effort and optimal performance, as shown in Figure 1. Optimal performance often refers to an effortless and automatic, flow-like state of performance. Mindfulness (mindful acceptance) regulates the focus of attention to optimal focus (balanced attention) on the core component of the action, avoiding too much attention that could be detrimental for elite performance (Bertollo et al., 2016). Balanced attention is a trained state that can optimize any particular attentional activity on the dual-process spectrum. One can exert minimal effort to maintain balanced attention, resulting in a large impact on performance in cognition, positive emotion, health and quality of life. To optimize tasks that require high effort and explicit processing such as working memory, one can reallocate attentional resources, resulting in more efficiently focused attention and less effort. To optimize tasks that require low effort and implicit processing such as creativity or sensorimotor skills, one can bring diffused attention to the task, resulting in more control and monitoring. Through balanced attention, different activities with different cognitive demands can be optimized with a balance of implicit and explicit processing, the appropriate level of attention and effort. Balanced attention has also been called the “being” state (Tang and Posner, 2009, 2014; Tang et al., 2015; Tang, 2017).

**Figure 1:**
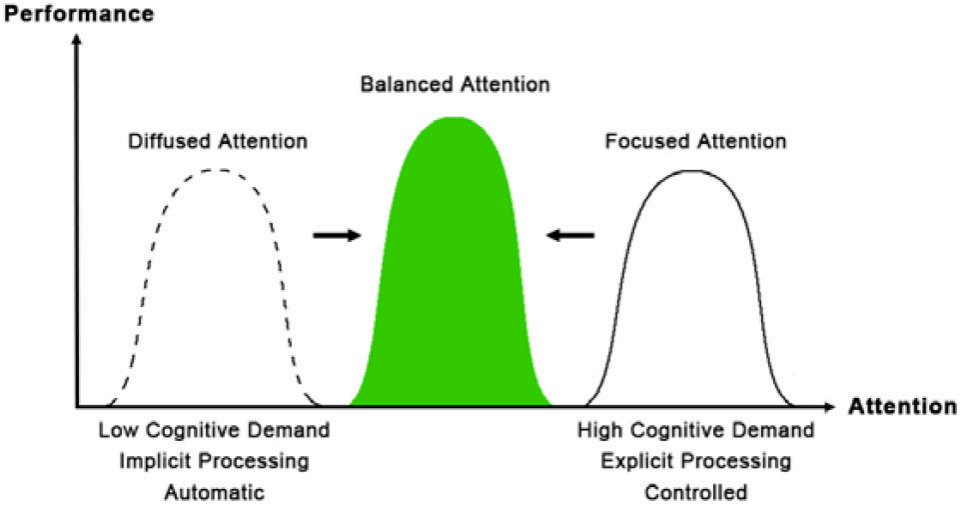
**Attention, effort, and optimal performance**. Balanced attention improves performance in both high effort, explicit processing tasks and low-effort, implicit processing tasks.

What are the underlying mechanisms supporting these distinct processes? Neuroimaging research has suggested that explicit processing with more effort, such as working memory tasks, often recruits the frontoparietal network (Takeuchi et al., 2010; Tang and Posner, 2009, 2014; Ekman et al., 2016; Nissim et al., 2017). The frontoparietal network mainly includes the lateral frontal and parietal cortex and supports continuous effort. It should be noted that it is impossible to maintain a steady and continuous effort because attention states are in constant fluctuation regardless of ongoing task demands (Petersen and Posner, 2012; Tang, 2017; Tang et al., 2017). Studies have shown that attentional lapses lead to poor performance on the task and are associated with midline frontal areas such as anterior cingulate cortex (ACC) (Adam et al., 2015; Chang et al., 2015). In contrast, implicit processing with less effort, such as creativity and sensorimotor tasks, also involves the ACC, insula and striatum (Tang and Posner, 2009, 2014; Ding et al., 2014a,b). The ACC is involved in monitoring and maintaining a state by reducing conflict with other states; the insula involves switching between states; and the striatum is associated with the reward experience and habitual responses to make state maintenance easier (Tang et al., 2012, 2015; Tang and Tang, 2013). Meanwhile, the ACC and insula also collaborate to support the role of the autonomic nervous system (ANS) in maintaining the effortless state, which has parasympathetic dominance indexed by lower skin conductance response (SCR) and greater high frequency heart rate variability (HRV) (Tang et al., 2009; Tang, 2017). In contrast, sympathetic dominance more often occurs in effortful processing that requires alertness and activation of the frontoparietal network (Tang et al., 2012, 2009; Tang and Posner, 2014). These findings are consistent with the results of optimal and suboptimal performance in sports (Bertollo et al., 2013, 2015).

Studies have elucidated the interaction and dynamics between the central nervous system (CNS) and ANS (Critchley et al., 2003; Tang and Posner, 2009; Tang et al., 2009; Critchley and Harrison, 2013; Tang, 2017). For example, we examined the brain and physiological changes at rest before, during, and after 5 sessions of IBMT and active control—relaxation training. During and after training, compared to the relaxation control, the IBMT group showed significantly greater parasympathetic activity in each of these measures including heart rate, respiratory amplitude and rate, HRV and SCR. During and after IBMT, differences in HRV and EEG power suggested greater involvement of the ANS. Imaging data showed greater ACC, striatum and insula activity in the IBMT group. Most importantly, frontal midline ACC theta was also correlated with high-frequency HRV, suggesting control by the ACC over parasympathetic activity (Tang et al., 2009; Tang, 2017). These results indicate that brief IBMT induces better regulation of the ANS by a midline ACC brain system, suggesting the interaction and coordination of body and mind following IBMT, a form of mindfulness that optimizes activities for maximal self-control, attention and efficiency with minimal effort (Tang, 2017). Other studies have shown that parasympathetic activity is associated with the flow state (de Manzano et al., 2010; Keller et al., 2011; Thomson and Jaque, 2011; Jacobs, 2014), a prime example of balanced attention in which high control is achieved with low subjective mental effort (Bruya, 2010). We call this mechanism “parasympathetic-attentional interaction” or “PA mind-body interaction.”

In summary, growing empirical evidence indicates that PA mind-body interaction often triggers optimal performance and is one possible mechanism for optimizing performance (Tang and Posner, 2009, 2014; Bruya, 2010; Tang et al., 2012; Tang, 2017). PA mind-body interaction can also have a large impact on positive emotion, health benefits, quality of life and self-growth. The field of body-mind practice is rapidly growing. However the majority of research focuses on health and behavior effects (and related brain changes) from training (Lutz et al., 2008; Tang et al., 2015). There has been less effort to scientifically investigate the underlying mechanisms (e.g., key biomarkers) of mind-body interaction and optimal performance when practitioners engage and maintain an effortless state. The current perspective aims to address this research gap. By integrating evidence from neuroimaging with evidence from physiology we propose the key brain markers in the ACC-insula-striatum network and the key physiological markers in the parasympathetic regulation of HRV and SCR. This effort will also shed light on how humans learn and practice physical and mental training effectively. Future studies can examine the relationship between PA mind-body interaction and short-term or long-term training such as mindfulness and its underlying mechanisms, using psychosocial, physiological, multimodal neuroimaging, and genetic methods.

## Author contributions

All authors listed, have made substantial, direct and intellectual contribution to the work, and approved it for publication.

### Conflict of interest statement

The authors declare that the research was conducted in the absence of any commercial or financial relationships that could be construed as a potential conflict of interest.
